# Whole-Body Physiologically Based Pharmacokinetic Modeling of GalNAc-Conjugated siRNAs

**DOI:** 10.3390/pharmaceutics17010069

**Published:** 2025-01-06

**Authors:** Emilie Langeskov Salim, Kim Kristensen, Erik Sjögren

**Affiliations:** 1 Department of Pharmaceutical Bioscience, Translational Drug Discovery and Development, Uppsala University, SE-75124 Uppsala, Sweden; emlv@novonordisk.com; 2 Department of Discovery PKPD & QSP Modelling, Novo Nordisk A/S, DK-2760 Måløv, Denmark; kkri@novonordisk.com

**Keywords:** siRNA, physiologically based pharmacokinetic modeling, PK-Sim, ASGPR, RISC, GalNAc

## Abstract

**Background/Objectives**: N-acetyl-galactosamine small interfering RNAs (GalNAc-siRNA) are an emerging class of drugs due to their durable knockdown of disease-related proteins. Direct conjugation of GalNAc onto the siRNA enables targeted uptake into hepatocytes via GalNAc recognition of the Asialoglycoprotein Receptor (ASGPR). With a transient plasma exposure combined with a prolonged liver half-life, GalNAc-siRNA exhibits distinct disposition characteristics. We aimed to develop a generic GalNAc-siRNAs whole-body physiologically based pharmacokinetic–pharmacodynamic (WB-PBPK-PD) model for describing the pharmacokinetic–pharmacodynamic (PK-PD) relationship and overall tissue distribution in the open-source platform Open Systems Pharmacology Suite. **Methods**: Model development was performed using published studies in mice leveraging the PK-Sim^®^ standard implementation for large molecules with added implementations of ASGPR-mediated liver disposition and downstream target effects. Adequate model performance was achieved across study measurements and included studies adopting a combination of global and compound-specific parameters. **Results**: The analysis identified significant compound dependencies, e.g., endosomal stability, with direct consequences for the pharmacological effect. Additionally, knowledge gaps in mechanistic understanding related to extravasation and overall tissue distribution were identified during model development. The presented study provides a generic WB-PBPK-PD model for the investigation of GalNAc-siRNAs implemented in a standardized open-source platform.

## 1. Introduction

RNA interference (RNAi) is a natural regulatory defense mechanism for invasion of exogenous genes but has also become a mechanism of drug action due to its target-specific knockdown of disease-related proteins [1]. An example of such drug modality is the small interfering RNA (siRNA), consisting of a sense strand and an antisense strand. The endogenous siRNA exerts targeted gene regulation in the cell cytosol by loading its anti-sense strand into a family of Argonaute 2 (Ago2) proteins and forms RNA-Induced Silencing Complex (RISC) [1]. In the cytoplasm, the anti-sense strand guides the RISC to its own complementary mRNA, and from a series of events, the RISC cleaves the warranted mRNA sequence, giving diminished protein translation. The target specificity of siRNAs makes them well-suited drug candidates for a variety of diseases, even what earlier has been considered to be untreatable [2].

However, endogenous siRNAs are fairly large molecules, with a molecular wight ranging from 13 to 22 kDa, and negatively charged biological molecules that are efficiently eliminated via endogenous nucleases. As a result, they suffer from poor drugability properties, like short circulation time in plasma and limited potential for passive cell membrane translocation. Several strategies to modify the siRNA duplex have successfully been developed to address these aspects. Enhanced stabilization chemistry (ESC) and Advanced ESC (ADV ESC) strategies, via addition of phosphorothioate linkages, amongst others, are used to increase the overall stability and prolong the duration of effect [3].

A successful strategy for increased liver tissue uptake is to target the Asialoglycoprotein Receptor (ASGPR), which is expressed abundantly by hepatocytes. ASGPR substrate specificity is achieved by direct conjugation of the multivalent tris N-acetylgalactosamine (GalNAc)3 to the sense strand of siRNA duplex (GalNAc-siRNA). For further details, see Nair et al. [4]. When recognized by the ASGPR receptor, the GalNAc-siRNA complex undergoes receptor-mediated endocytosis. After endosomal uptake of the GalNAc-siRNA-ASGPR complex, low pH value in the endosome facilitates spontaneous GalNAc-siRNA cleavage. Afterwards, the siRNA duplex sequestrates, and a fraction of the free anti-sense strand escapes endosomal degradation and is released intracellularly, where it loads into Ago2. Givosiran, GIVLAARI^®^, for the treatment of acute hepatic porphyria (AHP), was the first GalNAc-conjugated siRNA to reach market approval in 2019 [5]. Since Givosiran in 2019, three GalNAc-conjugated siRNAs have been marketed, Lumasiran, Inclisiran, and Vutrisiran [6], and several products are currently in late-stage development [7].

Although the overall understanding of the mechanisms of therapeutic GalNAc-siRNA have in-creased, many aspects of the pharmacokinetic–pharmacodynamic (PK-PD) relationship and systemic disposition remain to be understood. To address these questions, multiscale modeling approaches, e.g., physiologically based pharmacokinetic (PBPK) models, can be applied, especially since the short plasma circulations (<4 h in mouse) are a poor surrogate for GalNAc-siRNAs concentrations in the biophase (≥500 h in mouse) at the target and are not readily applicable for establishment of traditional PK-PD and dose–response relationships [3]. In 2021, Ayyar et al. published a minimal PBPK (mPBPK) model that aimed to characterize the disposition of GalNAc-siRNAs in several species [8]. The liver was described by higher details than other organs, including a mechanistic implementation of the ASGPR-mediated uptake process. However, empirical implementations of extravasation, unspecific tissue partitioning, and distribution to other organs were adopted to capture the observed rapid plasma disappearance and dynamics in liver tissue accumulation. A higher level of physiologically and mechanistically relevant representations of these processes would increase confidence in pre-clinical-to-clinical translations in the development of new siRNA modalities, i.e., describing extravasation via the two-pore formalism theory. The two-pore formalism theory anticipates that continued and fenestrated endothelia contain small (rs = 4.4 nm) and large pores (rs = 22.85 nm) through which molecules extravasate. Therapeutic GalNAc-siRNAs are highly charged duplexes of 21–23 nucleotides, approximately 7.5 nm in length [9,10,11,12] and approximately 1 nm in radii [12], and could consequently be subject to small pore extravasation resistance. The two-pore formalism for extravasation of large molecules has previously been implemented in the open-source whole-body PBPK (WB-PBPK) model PK-Sim, which has been evaluated for several species [13].

The aim of this study was to develop a more mechanistically and physiologically relevant generic whole-body physiologically based pharmacokinetic–pharmacodynamic (WB-PBPK-PD) model for GalNAc-siRNAs, including the two-pore formalism for extravasation, liver disposition via ASGPR, and endosomal trafficking, as well as mRNA silencing and downstream target protein effects, by leveraging established models. No WB-PBPK-PD model has previously been reported to our knowledge.

## 2. Methods and Materials

For model development and qualification, a data set from the literature was compiled via digitization (WebPlotDig-itizer, version 4.6). The data were extracted as mean values, so the estimated model parameters should be regarded as approximate and suitable for their intended purpose. For characterization on distribution, uptake, and elimination, data on GalNAc-siRNAs targeting antithrombin (ALN-AT3 (Fitusiran) [8,14] in (C57/129) mice and SIAT-2 [15]) in C57BL/6 mice, transthyretin protein (SITTR-1/SITTR-2) [15] in C57BL/6 mice, factor IX (siF9-1 and siF9-2) [4], and factor VII (siF7-1, siF7-2 and siF7-3) in C57BL/6 mice were used. Besides different target specificities, the Gal-NAc-siRNAs possessed two different stabilization designs, ESC and advanced ESC, allowing determination of com-pound-specific PBPK-PD parameters. The data further comprised liver mRNA levels for each compound included, as well as time-course data of the downstream effect on target protein for ALN-AT3 and SITTR-1. A liver tissue density of 1 mg/mL was assumed for converting the concentrations of Ago2-loaded sense strand and liver mRNA from weight-based to volume-based measurements. An overview of compounds and data included in this study, together with relevant information is summarized in Table 1. For further details on sample quantifications and analysis see respective references depicted in Table 1.

### 2.1. Model Development and Software

The presented WB-PBPK-PD model was developed and validated as an extension of the generic model for proteins and large molecules implemented in the open-source platform Open Systems Pharmacology Suite, PK-Sim^®^/MoBi^®^, Version 11.2 (https://www.open-systems-pharmacology.org/) [13]. The implementation involves the two-pore formalism for extravasation and elimination from vascular endothelial endosomes and lymphatic drainage while passive intracellular uptake was set to zero. In brief, this WB-PBPK-PD mode includes 15 individually represented tissue compartments connected by blood flows. Each tissue compartment is further subdivided into compartments representing the vascular space, interstitial space, endothelial endosomes, and intracellular space. Renal elimination was included as passive glomerular filtration parameterized according to the default implementation and the physiological database. Model optimization was accomplished by a “middle-out approach”, using in vivo/in vitro information combined with model parameter optimization via the Monte Carlo algorithm included in the software. Graphs and figures were created in R (http://www.r-project.org), R studio version 4.4.1 [16].

### 2.2. Extravasation Model

The exchange of GalNAc-siRNA between the plasma and the interstitial compartment in each organ represented in the model was described as an organ-specific flux rate, J__vi,org_ (amount per time), according to the two-pore formalism theory (Equation (1)) [13].

(1) $$J_{vi,org}=fu\cdot \left(\begin{array}{c} J_{L,org}\cdot \left(1-\sigma_{Lorg}\right)\cdot C_{vorg}+PS_{Lorg}\cdot \left(C_{vorg}-\frac{C_{iorg}}{K_{ivorg}}\right)\cdot \frac{{P_{e}}_{Lorg}}{e^{{P_{e}}_{Lorg}}-1}+\;\\ J_{S,org}\cdot \left(1-\sigma_{Sorg}\right)\cdot C_{vorg}+PS_{Sorg}\cdot \left(C_{vorg}-\frac{C_{iorg}}{K_{ivorg}}\right)\cdot \frac{{P_{e}}_{Sorg}}{e^{{P_{e}}_{Sorg}}-1} \end{array}\right)$$

In brief, J_vi_ depends on the plasma and interstitial concentration (C_v_ and C_i_), the transcapillary fluid flow rate (J), the fraction of unbound drug in plasma (fu), the partition coefficient between interstitial space and plasma (K_iv_), the reflection coefficients (σ), the Peclet number (P_e_), and the product of permeability and surface area (PS) for the large (L) and small (S) pores for in each organ (org). The pore permeability is calculated from the free diffusion coefficient of the solute, the ratios of the effective pore areas available for restricted diffusion through circular holes, the total cross sectional pore areas, the effective thickness of the endothelial membrane, and the capillary surface area. Further details on the implementation of large-molecule disposition in PK-Sim^®^ can be found in Niederalt et al., 2018 [13].

To support the two-pore formalism theory and characterize the fast extravasation of the GalNAc-siRNA in the liver tissue, an organ-specific permeability in the liver, P_liver_, was implemented as described in Equation (2):

(2) $$\frac{dC_{int}}{dt}=fu\cdot P_{liver}\cdot SA_{liver}\cdot \left(C_{pls}-\frac{C_{int}}{Kp}\right)$$

where fu is the fraction of unbound drug in plasma, P_liver_ is the organ-specific permeability, SA_liver_ is the liver surface area, C_pls_ is the concentration of drug in plasma, C_int_ is the concentration of drug in the interstitial space, and Kp is the partition coefficient of the drug between plasma and interstitial space.

### 2.3. ASGPR-Mediated Uptake by TMDD Model

Specification of siRNA and ASGPR/RISC dynamics, e.g., binding, internalization, recycling, and downstream translation, was further implemented in MoBi by a target-mediated drug disposition (TMDD)-model approach. The TMDD model was originally developed by Kzryzanski et al. [17] and further modified by Ayyar et al., 2021, who implemented partitioning of GalNAc dissociation from the receptor in the endosome, recycling of the free receptor from the endosome to the cell surface, endosomal receptor degradation, and escape of siRNA loading into Ago2 and form RISC [8]. In the current model, Equations (3)–(5) were used to calculate the kinetics in the free receptor concentration in the interstitial compartment (A_free_), accounting for the recycling of the receptor to the cell surface membrane (Equation (3)), the concentration of the free GalNAc-siRNA in the interstitial compartment (D_free_; Equation (4)), and the endosomal concentration of the ASGPR-GalNAc-siRNA complex (DA; Equation (5)).

(3) $$\frac{dA_{free}}{dt}=k_{syn}-k_{deg}\cdot A_{free}-k_{on}\cdot D_{free}\cdot A+k_{off}\cdot DA+k_{rec}\cdot A_{endosme}$$

(4) $$\frac{dD_{free}}{dt}=-k_{on}\cdot D_{free}\cdot A_{free}+k_{off}\cdot DA$$

(5) $$\frac{dDA}{dt}=k_{on}\cdot D_{free}\cdot A_{free}-k_{off}\cdot DA-k_{int}\cdot DA$$

where k_syn_ is the zero-order synthesis rate constant of the ASGPR, k_deg_ is the first-order elimination rate constant of ASGPR, k_on_ is the first-order binding rate constant, k_off_ is the first-order dissociation rate constant of the GalNAc-siRNA, k_int_ is the first-order internalization rate constant (elimination of the drug-receptor complex), and k_recycle_ is the first-order recycle rate constant of the free receptor concentration in the endosome (A_endosome_) that recycles back to the cell membrane. ASGPR-mediated uptake parameters were based on values reported from Ayyar et al., 2021 [8] and were fixed prior to simulation or estimated during simulation, as depicted in Table 2. For the full TMDD model description, see the Supplementary Materials, and for the full model, see Supplementary Materials Model File: WB-PBPK-PD_ALN-AT3_1 mg_kg_Case_Example.

### 2.4. Pharmacodynamics

The endogenous homeostasis of the drug-response variables, mRNA, and target protein, was described by a turnover model characterized by a zero-order synthesis rate constant, k_syn_, and a first-order degradation rate constant, k_deg_. Thus, the following initial condition would be implemented:

(6) $$k_{syn.mRNA}=k_{deg.mRNA}\cdot mRNA_{0}$$

(7) $$k_{syn.protein}=k_{deg.Protein}\cdot Protein_{0}$$

As the pharmacodynamics were described as relative change to baseline, the absolute value for mRNA_0_ and Protein_0_ was set to 100%. As a consequence, k_syn_ and k_deg_ correlated with a fixed factor of 100.

The pharmacodynamics of RISC-induced mRNA silencing and target protein knockdown was described as relative change from baseline (mRNA_0_ and Protein_0_). The rate of RISC-induced mRNA silencing was modeled with an indirect stimulatory response model (IDR) (Equation (5)).

(8) $$\frac{dmRNA}{dt}=k_{deg.mRNA}\cdot \left(mRNA_{0}-\left(1+\frac{S_{max}\cdot CRISC}{SC_{50}+CRISC}\right)\cdot mRNA\right)$$

where k_deg.mRNA_ represents the degradation rate constant of the target mRNA; mRNA_0_ is the baseline value of mRNA equal to 1; CRISC is the concentration of the siRNA-induced RISC; S_max_ describes the maximum effect induced; and SC_50_ is the concentration required to produce half the maximum effect. The knockdown of target protein was regulated in response to the mRNA level relative to mRNA baseline (mRNA_0_) (Equation (9)):

(9) $$\frac{dProtein}{dt}=k_{deg.protein}\cdot \left(Protein_{0}\cdot {\left(\frac{mRNA}{mRNA_{0}}\right)}^{y}-Protein\right)$$

where k_deg.protein_ represents the degradation rate constant of the target protein, and γ is an empirical power function describing the slope of the response to the change in mRNA levels.

### 2.5. Model Strategy and Assumptions

The general model strategy was to leverage the previously described WB-PBPK model for large molecules and the ASGPR TMDD model to establish a WB-PBPK-PD model [8]. First, PK-Sim^®^ was used to establish the generic structure for the WB-PBPK model for a GalNAc-siRNA molecule. The “model for proteins and large molecules” was selected to include the size dependent two-pore formalism for extravasation and endothelial endosomal clearance. A first-order input function to central venous plasma was included to simulate systemic drug appearance after intravenous (IV) and subcutaneous (SC) drug administration. The PBPK model was exported to MoBi^®^, where additional elements were added to accommodate the investigation of general disposition processes, ASGPR-mediated liver uptake, and downstream PD effects. The generic implementation for endosomal clearance in PK-Sim^®^, calculated as the difference between endosomal uptake and recycling rate, was disabled and replaced with a first-order metabolic reaction in plasma and all organs, except the liver. The ASGPR system was added to the endosomal sub-compartment in the liver compartment with uptake from the interstitial compartment and endosomal escape to the intracellular compartment. Initial parameter values were informed by compound characteristics and the default settings in PK-Sim^®^, and they were as reported in the ASGPR studies [19,20,21,22,23]. In accordance with previous investigations, the influence of plasma protein binding (PPB) on disposition processes was assumed to be negligible [24]. An iterative model development strategy was then applied comparing simulation output to compiled reference data. The approach adopted an iterative increase in deviation from the legacy models and flexibility in terms of compound-specific parameter estimation. Compound differences were also expected. For example, the degradation rate may differ between the GalNAc-siRNA sequence and chemical stabilization method, which also may influence effective bioavailability after SC administration [25]. Likewise, endosomal degradation and endosomal release into the cytoplasm are believed to be sequence/formulation-specific [3,26].

Uptake into cells was assumed to be mediated by ASGPR and was hence restricted to the liver [23]. The ASGPR PK parameters were adopted from Ayyar et al., 2021 [8] or optimized when appropriate; see Table 1. Equally, it was assumed that the receptor kinetics is mainly driven by the GalNAc ligand, except for the internalization rate of the ASGPR-GalNAc-siRNA complex that was assumed to be dependent on the physiochemical properties of the siRNA [27]. We anticipated that 1% of the unconjugated siRNA would escape into the cytoplasm to induce RISC formation and exhibit mRNA cleavage [25]. Further, it was anticipated that the fraction of cleaved mRNA and the time interval before mRNA is fully reestablished would depend on the RISC formation but that the dissociation from the RISC will not influence the PK-PD relationship [3,15].

Because of incomplete data, certain processes had to rely on a subset of compounds, assuming their similarity across all. Systemic, kidney, and liver disposition processes, as well as the link between these, were established by compounds with plasma-, kidney-, and liver-tissue data. The fraction escaping endosomal degradation and siRNA-RISC complex kinetics was informed by compounds with liver tissue and siRNA-RISC complex measurements. Compounds with siRNA-RISC, mRNA, and downstream protein expression were used to inform the PD. The pharmacodynamics were characterized after the establishment of the siRNA disposition and siRNA-RISC complex dynamics. Investigated compounds with respective available data are summarized in Table 1.

### 2.6. Model Evaluation

The model was subject to a sensitivity analysis to identify PBPK-specific sensitive parameters impacting the PK and PD. The sensitivity analysis was conducted according to PK-Sim^®^ [28] and performed in R (http://www.r-project.org), R studio [16]. The sensitivity analysis calculates the relative impact for a given PK parameter to changes in different input parameters with a variation of 10%. The sensitivity of a PK parameter was calculated as the ratio of the relative change in the PK parameter and the relative variation in the input parameter, as in Equation (10):

(10) $$S_{ij}=\frac{∆PK_{j}}{∆p_{i}}\cdot \frac{p_{i}}{PK_{j}}$$

where ∆PK_j_ is the change in the PK parameter, ∆p_i_ is the change in the input parameter, PK_j_ is the value of the PK parameter, and p_i_ is the value of the input parameter. According to the WHO’s guidelines, sensitivities were evaluated as being high (absolute value ≥ 0.5), medium (absolute value ≥ 0.2 but less than 0.5), or low (absolute value ≥ 0.1 but <0.2) [29]. Parameters with a sensitivity < 0.1 were considered to have an insignificant influence on the evaluated PK parameter. The PK parameter evaluated was exposure, calculated as area under the curve simulated from time 0 h to 1000 h (AUC_0–1000h_) via the R package ncappc (CRAN: Package ncappc), in R studio [16].

The overall model performance was evaluated quantitatively by calculating the geometric mean of the ratio between the absolute simulated area under the curve (AUC_sim_) and the observed area under the curve (AUC_obs_) estimate as the absolute average fold error (AAFE), along with the average fold error (AFE); see Supplementary Materials for further details. The model’s performance was deemed adequate, with 0.5 ≤ AFE ≤ 2 and AAFE ≤ 2, taking into account the variability and sparsity of the compiled data, as well as potential differences in bioassay methodologies across studies.

## 3. Results

Overall, the WB-PBPK-PD successfully characterized the PK-PD relationship of GalNAc-siRNAs. Furthermore, the presented model identified compound-specific PBPK parameters and shed light on the knowledge gaps of the GalNAc-siRNA biodistribution.

### 3.1. Characterization of Extravasation via Two-Pore Formalism

The generic two-pore formalism could not be readily employed to describe the extravasation of the investigated GalNAc-siRNAs. By this parameterization, the molecules were restricted to plasma (see Supplementary Figure S1), thus prolonging the circulation time, while limiting ASGPR-mediated uptake and failing to capture the fast onset in liver concentrations. To satisfactorily characterize the extravasation of GalNAc-siRNAs by the two-pore formalism, an empirical permeability, P_liver_, was introduced and estimated via Monte Carlo simulation to have a value of 0.02 cm/min.

### 3.2. Characterization of General Tissue Distribution and Plasma Concentration

**Figure 1:**
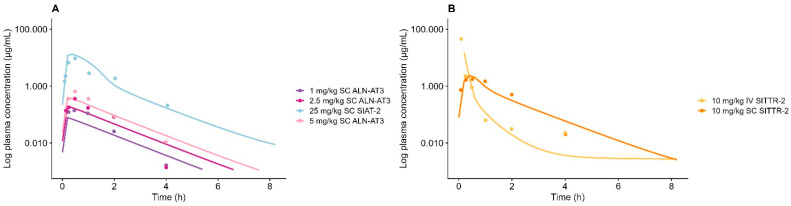
Model-simulated plasma concentration profiles vs. observed data for GalNAc-siRNAs (**A**) targeting antithrombin (ALN-AT3/SIAT-2) and (**B**) targeting transthyretin protein (SITTR-2). Solid lines represent model simulations, and dots represent observations. (**A**) Dark purple line represents 1 mg/kg of subcutaneously administered ALN-AT3, dark pink represents 2.5 mg/kg of subcutaneously administered ALN-AT3, light pink line represents 5 mg/kg of subcutaneously administered ALN-AT3, and light blue line represents 25 mg/kg of subcutaneously administered SIAT-2. (**B**) Orange line represents 10 mg/kg of subcutaneously administered SITTR-2, and yellow line represents 10 mg/kg of intravenously administered SITTR-2.

Overall, the model successfully characterized the short half-life of the plasma concentrations. The observed plasma concentrations and simulation are presented in Figure 1. To accurately describe plasma PK, the model identified a necessity to introduce general tissue distribution, in this case, accommodated for as unspecific first-order endosomal uptake from and recycling to interstitial. Uptake and recycling rate constants (k_uptake_ and k_recycle_) were globally estimated to be 20.9 min^−1^ and 7.72 × 10^−5^ min^−1^, respectively. The ratio of plasma AUC_sim_/AUC_obs_ was estimated within 2-fold (0.52–1.22); see Supplementary Table S1 for further details. The AFE was estimated to have a value of 0.73, which is within the range for adequate model performance. The AAFE was estimated, according to the criteria for successful model performance, to have a value of 1.47. The model further identified a shared plasma degradation rate, k_RNase_, of 0.00012 h^−1^ and a shared subcutaneous absorption rate of 0.83 h^−1^. The bioavailability (F) was estimated as a compound-specific parameter across doses, except for SIAT-2, for which a dose-specific F for the highest dose (25 mg/kg) was needed in order to capture observations; see Table 3.

### 3.3. Recalibration of TMDD Model Parameters

Some recalibrations of the previously reported TMDD ASGPR model parameters were performed as well. To accurately describe the ASGPR-mediated uptake in the non-linear spectrum (dose ≥ 10 mg/kg), the receptor density (R_tot_), the receptor turnover (k_deg_), and internalization rate constant of complex-bound ASGPR-GalNAc-siRNA (k_int_) were all estimated during Monte Carlo parameter optimization to be values of 5.23 umol/L, 1.52 h^−1^, and 5.14, respectively. See Table 2 for all ASGPR-related parameters and how they were achieved.

### 3.4. Characterization of Liver-Tissue Concentrations

**Figure 2:**
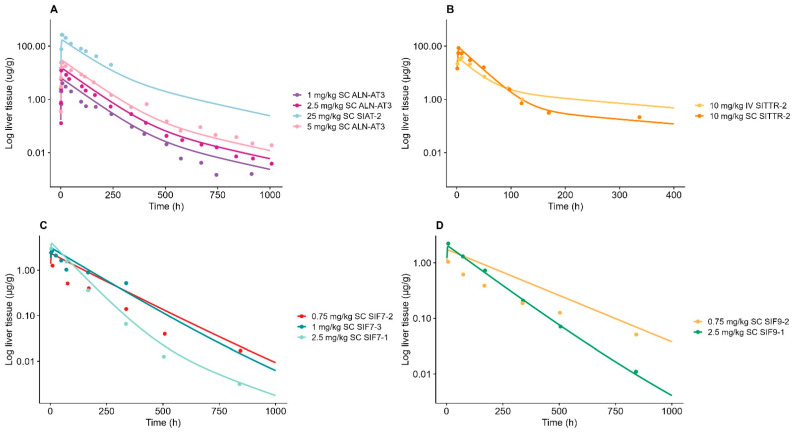
Model-simulated liver tissue distribution vs. observed data for GalNAc-siRNAs (**A**) targeting antithrombin (ALN-AT3/SIAT-2), (**B**) targeting transthyretin protein (SITTR-2), (**C**) targeting factor 7, and (**D**) targeting factor 9. Solid lines represent model simulations, and dots represent observations. (**A**) Dark purple line represents 1 mg/kg of subcutaneously administered ALN-AT3, dark pink represents 2.5 mg/kg of subcutaneously administered ALN-AT3, light pink line represents 5 mg/kg of subcutaneously administered ALN-AT3, and light blue line represents 25 mg/kg of subcutaneously administered SIAT-2. (**B**) Orange line represents 10 mg/kg of subcutaneously administered SITTR-2, and yellow line represents 10 mg/kg of intravenously administered SITTR-2. (**C**) Red line represents 0.75 mg/kg of subcutaneously administered SIF7-2, blue line represents 1 mg/kg of subcutaneously administered SIF7-3, and light green line represents 2.5 mg/kg of subcutaneously administered SIF7-1. (**D**) Light orange line represents 0.75 mg/kg of subcutaneously administered SIF9-2, and green line represents 2.5 mg/kg of subcutaneously administered SIF9-1.

During model development, the endosomal degradation/escape rate (k_endosome_) was found to be highly influential on the elimination phase (250–1000 h) of the liver-tissue concentration. The elimination phase in the liver was found to be compound-specific, as depicted in Figure 2, portraying the normalized liver tissue-concentration dose. By individually estimating the k_endosome_ value for each compound, the TMDD ASGPR model accurately described the liver half-life across all compounds, as shown in Figure 2. All the liver AUC_sim_/AUC_obs_ ratios were estimated within 2-fold (0.82–1.86); see Supplementary Table S1. The AFE and AAFE for the liver concentration were estimated, within the criteria of adequate model performance, to be 1.10 and 1.34, respectively. As expected, we observed that GalNAc-siRNA formulated with Adv ESC showed a tendency towards a lower endosomal degradation rate compared to its corresponding GalNAc-siRNAs formulated with ESC instead; see Table 2 for further details. The fraction escaping endosomal degradation (f_escape_) and intracellular degradation in the cytoplasm k_cytoplasm_ was fixed to a value of 1% and 0.1 h^−1^, respectively, to avoid overparameterization [8].

### 3.5. siRNA-Induced RISC Formation

**Figure 3:**
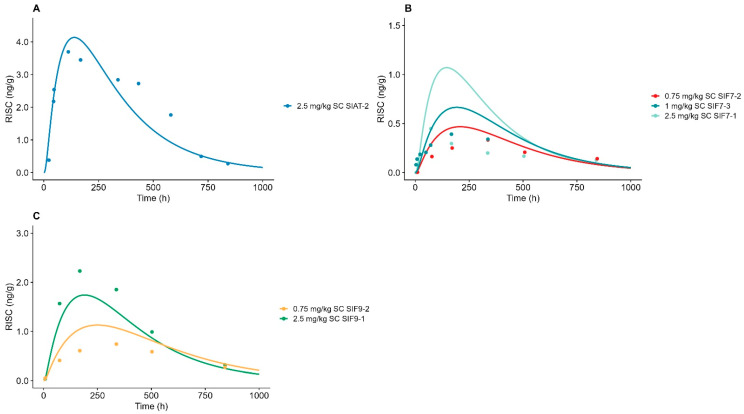
Model-simulated siRNA-induced RISC formation vs. observed data measured as antisense strand loaded into Ago2 for GalNAc-siRNAs targeting (**A**) antithrombin (SIAT-2), (**B**) factor 7 (siF7-1/2/3), and (**C**) factor 9 (siF9-1/2). Solid line represents model simulation, and dots represent observed data points. (**A**) Dark blue line represents 2.5 mg/kg of subcutaneously administered SIAT-2. (**B**) Red line represents 0.75 mg/kg of subcutaneously administered SIF72, blue line represents 1 mg/kg of subcutaneously administered SIF73, and light green line represents 2.5 mg/kg of subcutaneously administered SIF7-1. (**C**) Light orange line represents 0.75 mg/kg of subcutaneously administered SIF9-2, and green line represents 2.5 mg/kg of subcutaneously administered SIF9-1.

Overall, the ASGPR TMDD model succeeded in describing the siRNA-induced RISC formation, as shown in Figure 3. The ratios of the AUC_sim_/AUC_obs_ were all estimated within 2-fold, except for siF7-1 2.5 mg/kg, with an AUC_sim_/AUC_obs_ ratio of 2.44; see Supplementary Table S1. The AFE and AAFF were 1.32 and 1.47, indicating a minor trend of overprediction in the RISC formation, but both the AFE and AAFE were within the limitations of successful model performance. The k_DR_ and kon__RISC_ were globally estimated to be 0.0045 1/h and 0.00027 nmol/L/h, respectively, except for sif9-1/2, which was described by an individual k_onRISC_ estimate of 0.0014 nmol/L/h.

### 3.6. Characterization of Kidney Tissue Distribution

**Figure 4:**
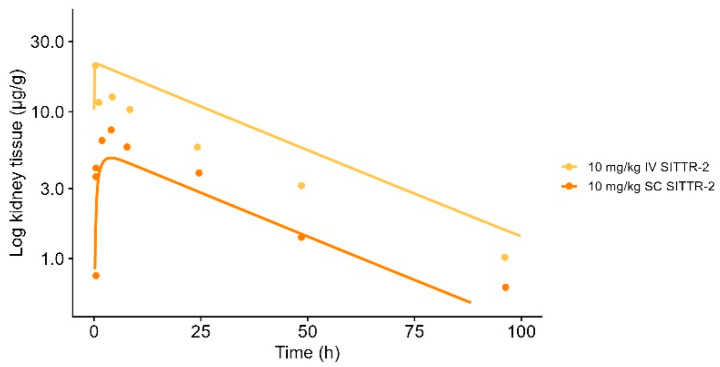
Model-simulated kidney tissue distribution vs. observed data for GalNAc-siRNA targeting transthyretin protein (SITTR-2). Solid line represents model simulation, and dots represent observed data points. Orange line represents 10 mg/kg of subcutaneously administered SITTR-2, and yellow line represents 10 mg/kg of intravenously administered SITTR-2.

The characterization of the kidney distribution was informed by the data from GalNAc-siRNAs targeting transthyretin protein, SITTR-2 10 mg/kg (SC and IV). The kidney concentrations were captured successfully, as shown in Figure 4, with AUC_pred_/AUC_obs_ ratios estimated within 2-fold (1.42–0.64) for the SC and IV administration, respectively. The AFE was estimated to have a value of 0.95, and the AFFE value was estimated to be 1.49. Distribution kinetics into renal tissue were described by unspecific endosomal trafficking (k_kid.uptake_ = 68.6 min^−1^ and k_kid.recycle_ = 3.19 × 10^−4^ min^−1^). Renal elimination was implemented as passive filtration of unbound drug via the glomerular filtration rate into urine.

### 3.7. PK-PD Relationship

**Figure 5:**
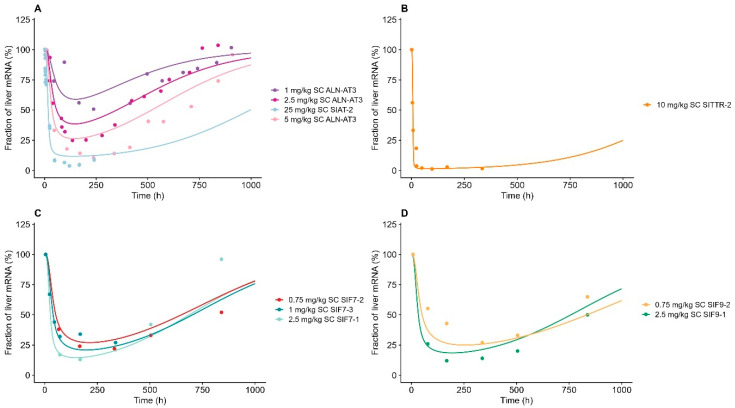
Model-simulated percentage liver mRNA silencing vs. observed data for GalNAc-siRNAs (**A**) targeting antithrombin (ALN-AT3/SIAT-2), (**B**) targeting transthyretin protein (SITTR-2), (**C**) targeting factor 7 (siF7-1/2/3), and (**D**) targeting factor 9 (siF9-1/2). Solid line represents model simulation, and dots represent observed data points. (**A**) Dark purple line represents 1 mg/kg of subcutaneously administered ALN-AT3, dark pink represents 2.5 mg/kg of subcutaneously administered ALN-AT3, light pink line represents 5 mg/kg of subcutaneously administered ALN-AT3, and light blue line represents 25 mg/kg of subcutaneously administered SIAT-2. (**B**) Orange line represents 10 mg/kg of subcutaneously administered SITTR-2. (**C**) Red line represents 0.75 mg/kg of subcutaneously administered SIF72, blue line represents 1 mg/kg of subcutaneously administered SIF7-3, and light green line represents 2.5 mg/kg of subcutaneously administered SIF7-1. (**D**) Light orange line represents 0.75 mg/kg of subcutaneously administered SIF9-2, and green line represents 2.5 mg/kg of subcutaneously administered SIF9-1.

**Figure 6:**
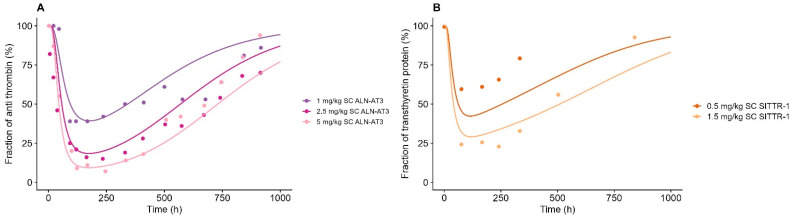
Model simulations of the percentage downstream effect on target protein vs. observed data measured as serum antithrombin and serum transthyretin for GalNAc-siRNAs (**A**) targeting antithrombin (ALN-AT3/SIAT-2) and (**B**) targeting transthyretin protein (SITTR-1). Solid line represents model simulation, and dots represent observed data points. (**A**) Dark purple line represents 1 mg/kg of subcutaneously administered ALN-AT3, dark pink represents 2.5 mg/kg of subcutaneously administered ALN-AT3, and light pink line represents 5 mg/kg of subcutaneously administered ALN-AT3. (**B**) Dark orange line represents 0.5 mg/kg of subcutaneously administered SITTR-1, and yellow line represents 1.5 mg/kg of subcutaneously administered SITTR-1.

The gene-silencing effect of the target mRNA and downstream effect of the target were successfully characterized by an indirect response model, as depicted in Figure 5 and Figure 6, respectively. The estimates of the PD parameters previously estimated for ALN-AT3 were initially applied to the model [8]. Yet S_max_ and SC_50_ required optimization across all compounds to fully capture observations, but k_deg.mRNA_ and k_deg.protein_ were fixed to previously published values; see Table 3 [7]. The S_max_ and SC_50_ were estimated individually for each compound, except SC_50_, for which sif7-1/2/3 and sif9-1/2 were estimated as a shared parameter. Overall, the SC_50_ had the highest variation between compounds with a 10-fold variation, going from 13.05 to 140.15, whereas Smax had a 2-fold variation, going from 1.71 to 4.06, with SIITR-2 depicting the highest value of S_max_ (140.05) and SC_50_ (4.06). For a PBPK-PD parameter overview, see Table 3.

The downstream effect on the target protein was captured well for ALN-AT3 and SITTR-2 based on the response of the mRNA levels uplifted with an estimated empirical power function, gamma, with a value of 1.77 for ALN-AT3 and 0.42 for SITTR-2.

### 3.8. Sensitivity Analysis

**Figure 7:**
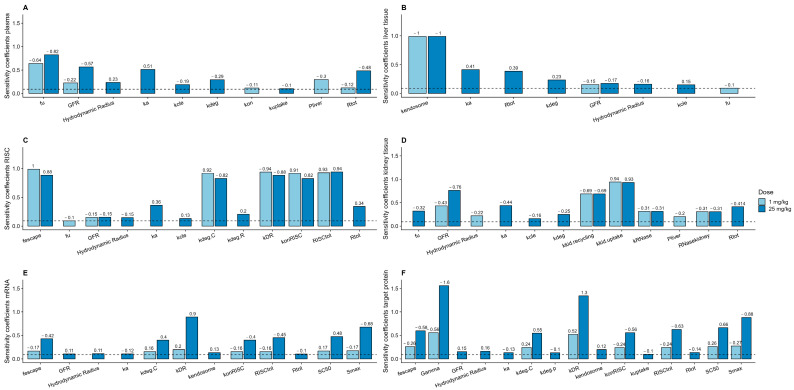
Model sensitivity analysis of PBPK parameters measured as the change in area under the curve (AUC) (ug·h/mL) simulated from time 0 h to time 1000 h (AUC_0–1000h_) for 1 mg/kg (light blue bars) and 25 mg/kg (dark blue bars) in (**A**) plasma, (**B**) liver tissue, (**C**) siRNA-induced RISC, (**D**) kidney tissue, (**E**) mRNA silencing, and (**F**) downstream effect on target protein. Positive sensitivity coefficient denotes an increase in AUC_0–1000h_ when the investigated parameter is increased by 10%. Negative sensitivity coefficients denote a decrease in AUC_0–1000h_ when the investigated parameter is increased with 10%. Dashed line signifies the threshold of 0.1 used to assess the minimum impact of the PBPK parameter.

Local parameter sensitivity analysis was performed towards exposure (AUC_0–1000h_) for the model readouts: plasma concentration, liver-tissue concentration, RISC, kidney tissue concentration, and mRNA silencing, as well as effect on target protein. To examine potential dose dependencies, the analysis was conducted for both a low and high dose, 1 mg/kg and 25 mg/kg, representing the dose range of the included reference studies. Clear dose and cross-process dependencies were identified for all included model readouts, with a higher overall sensitivity for the high dose (25 mg/kg) investigated (Figure 7). At the low dose (1 mg/kg), plasma and liver exposure were mainly sensitive to fu and k_deg_, respectively, while additional parameters, e.g., ka, GFR, and R_tot_, also were influential at the high dose (25 mg/kg; Figure 7A,B). Kidney exposure showed the highest sensitivity to kidney distribution parameters (k_kid.uptake_ and k_kid.recycling_) and GFR (Figure 7D). siRNA-induced RISC, mRNA silencing, and downstream effect on target protein showed sensitivity to multiple parameters directly related to these processes, as well as to siRNA disposition processes upstream (Figure 7C,E,F).

## 4. Discussion

GalNAc-siRNAs with selective silencing of disease-related proteins and target-specific uptake in the liver are, to this date, the most successful RNA drug modality [1]. In this study, we present a generic WB-PBPK-PD model for Gal-NAc-siRNAs describing plasma PK, ASGPR-mediated liver disposition and PD. The model was informed via observation of 10 GalNAc-siRNAs in mice and leveraged previously established models [8,13].

Overall, the presented WB-PBPK-PD model characterized the PK-PD relationship well across all compounds. The final model adopts a generic structure and parameterization according to the legacy WB-PBPK model [13] and ASGPR TMDD model [8], with only a few modifications and compound-specific parameters. The presented study also highlights the remaining knowledge gaps related to the drugs’ distribution. We identified that the generic two-pore formalism could not adequately explain liver extravasation of GalNAc-siRNAs, as it restricted them to plasma and underestimated liver accumulation. The need to manipulate the two-pore formalism has been observed previously in the literature to describe enhanced permeability of mAbs in tumor tissue [30]. This approach was investigated; however, it did not satisfactorily predict the PK, and liver tissue-exposure alterations beyond plausibility were needed; see the Supplementary Materials for model simulations with generic implementations. An additional generic pathway for extravasation was therefore implemented for this organ in addition to the default two-pore formalism. One theoretical explanation for this could be an interaction between the GalNAc linker and the negatively charged glycocalyx, a sugar coat attached to the apical surface of vascular endothelial cells. It has been shown that glycocalyx, mimicking nanoparticles, preferably absorbs into different tissues, likely due to interaction with other sugar moieties from the glycocalyx [31]. To enhance entry into cell hosts interactions between different sugar moieties (glycans, glycolipids, and glycoproteins) and glycocalyx have also been observed for human immunodeficiency virus (HIV) to enhance its entry into cell hosts [32]. Another potential explanation could be RNA-induced vessel permeability via elevation of flux and disintegration of tight junctions, as it is supported by the accumulation of unconjugated siRNAs in intestines, gland tissue, etc., in addition to the liver, indicating that the enhanced extravasation process is, to some extent, regulated by basal functionalities [33,34,35]. In summary, this study highlights the importance of RNA/siRNA/GalNAc-siRNA extravasation for general- and liver-tissue accumulation, and further insights into this process may open new possibilities for tissue targeting.

The final model was able to accurately describe the liver-tissue concentrations with high precision, with an AFE of 1.10 and an AAFE of 1.34, as well as AUC_sim_/AUC_obs_, all within a 2-fold. Saturation of ASGPR-mediated uptake, which limited liver exposure, was described by the model at higher doses (>5 mg/kg), in accordance with observations. Model simulations were comparable to the reported simulations published in the legacy model, while simulations for newly introduced measurements for higher doses, i.e., liver-tissue concentration of SITTR-2 10 mg/kg SC/IV, required optimization to capture non-linearities. A few parameters, such as the receptor density (R_tot_) and internal k_int_ and k_deg_, were estimated for optimal performance, which is not unexpected given the difference in model structure and additional data included. The need to increase receptor turnover (k_deg_) has also been observed in other previously published PBPK models [36]. The receptor density R_tot_ and internal k_int_ and k_deg_ used in the legacy model was originally determined via a murine model, testing the liver uptake of IV administered GalNAc–interleukin-2 (1–30 mg/kg) [18]. Recently published literature indicates that the effective receptor activity used as a proxy to determine abundance is not solely dependent on the GalNAc linker; it is also dependent on the physicochemical properties of the modality attached to the GalNAc [27,37]. For instance, in vitro experiments have shown that a hydrophilic linker on GalNAc, such as siRNA molecules, tends to have a lower steric hindrance for effective ASGPR recognition and increased uptake for up to 30% compared hydrophobic linkers such as IL-2 [27,38]. Second, other studies indicate that the composition of the nucleotides and the number of phosphor bindings on the backbone of the siRNA duplex may also influence receptor affinity [37]. The need for increased receptor density and turnover could also be explained by compensation from other glycan binding receptors, such as the mannose receptor C-type 1 (MRC1), located on the sinusoidal endothelial cells [39]. At last, additional liver uptake from ASGPR was expressed specifically on mice macrophage Kupffer cells [40]. However, all the abovementioned hypotheses require further research.

The model further provides characterization of RISC formation, mRNA silencing and downstream effect of target protein. To provide as general a description as possible, global recalibration of the RISC dynamics was applied by optimization of k_DR_ and association rate constant k_onRISC_. Adequate performance was achieved with this approach although some cases showed less accuracy, e.g., overprediction of siF7-1 at 2.5 mg/kg (AUC_pred_/AU_Cobs_ = 2.44). The RISC dynamics was highly sensitive to k_DR_, and an improved performance could potentially be achieved by compound-specific estimations of this parameter [26]. However, to further inform the model implementation and gain insights into potential compound differences, additional knowledge and experimental investigations of the RISC creation process would be needed. Finally, RISC induced mRNA silencing, and the downstream effect of target protein was described by compound-specific parameters, anticipating differences in target protein dynamics and the empirical effect models applied. Interestingly, equivalent RISC SC_50_ values were identified across compounds (1.7–4.1 nmol/L), indicating that the potency of RISC mRNA silencing may be generalized, although mRNA and protein dynamics may differ depending on the target protein.

## 5. Conclusions

We present a generic GalNAc-siRNA WB-PBPK-PD model implemented in the open-source platform MoBi^®^. This comprehensive model encompasses all necessary elements to describe physiologically based systemic and liver disposition, including ASGPR-mediated liver uptake and RISC-induced mRNA silencing and protein reduction. As a result, the model is readily applicable for characterizing novel GalNAc-siRNAs in drug development and for hypothesis-driven investigations to elucidate specific disposition mechanisms. This model thus serves as a powerful tool for more effective development of GalNAc-siRNA therapeutics, ultimately advancing new RNAi treatments for patients.

## Figures and Tables

**Table 1:** Summary of the published data on the GalNAc-siRNAs and their measurements included in the model development of the presented WB-PBPK-PD model.

Compound	Target	Design	Administration/Dose	Measurement	Reference
ALN-AT3 (Fitusiran phase III)	Antithrombin	ESC ^a^	SC ^b^ 1–5 mg/kg	Plasma, liver, liver mRNA, serum antithrombin	Sehgal et al., 2015 [14]; Nair et al., 2017 [15]
SIAT-2 (investigational)	Antithrombin	Assumed ESC ^a^	SC ^b^ 2.5–25 mg/kg	Plasma, liver, liver mRNA, RISC ^d^	Nair et al., 2017 [15]
siF7-1 (investigational)	Coagulation Factor VII	ESC ^a^	SC ^b^ 2.5 mg/kg	Liver, liver mRNA, RISC ^d^	Brown et al., 2020 [3]
siF7-2/siF7-3 (investigational)	Coagulation Factor VII	Advanced ESC ^a^	SC ^b^ 0.75, 1 mg/kg	Liver, liver mRNA, RISC ^d^	Brown et al., 2020 [3]
siF9-1 (investigational)	Coagulation Factor IX	ESC ^a^	SC ^b^ 2.5 mg/kg	Liver, liver mRNA, RISC ^d^	Brown et al., 2020 [3]
siF9-2 (investigational)	Coagulation Factor IX	Advanced ESC ^a^	SC ^b^ 0.75 mg/kg	Liver, liver mRNA, RISC ^d^	Brown et al., 2020 [3]
SITTR-1/SITTR-2 (investigational)	Transthyretin protein	ESC ^a^	SC ^b^ 0.5, 1.5 mg/kg SC ^b^, IV ^c^ 10 mg/kg	Plasma, liver, liver, mRNA (SC), serum transthyretin protein	Nair et al., 2017 [15]; Brown et al., 2020 [3]

^a^ Enhanced stabilization chemistry; ^b^ subcutaneous; ^c^ intravenous; ^d^ RNA-Induced Silencing Complex.

**Table 2:** Summary of the ASGPR TMDD model parameters and global WB-PBPK-PD parameter values and their references.

ASGPR TMDD Parameters			
Parameter (Unit)	Description	Value	Reference
R_tot_ (μmol/L)	Total ASGPR density	5.23	Optimized
k_on_ (L/nmol/h)	Association rate constant between GalNAc-siRNA and ASGPR	0.53	Ayyar et al., 2021 [8]
k_off_ (h^−1^)	Dissociation rate constant between GalNAc-siRNA and ASGPR	1.53	Sato et al., 2002 [18]
k_deg·R_ (h^−1^)	Degradation rate constant of ASGPR in cytoplasm	1.53	Schwartz et al., 1982 [19]
k_deg_ (h^−1^)	Degradation rate constant of ASGPR on hepatocyte	1.52	Optimized
k_syn_ (h^−1^)	Synthesis rate constant of ASGPR	7.94	k_syn_ =R_tot_ · k_deg_
k_int_ (h^−1^)	Internalization rate constant of GalNAc-siRNA-ASGPR complex	5.14	Optimized
k_cle_ (h^−1^)	Cleavage rate constant of GalNAc-siRNA in endosome	1.32	Prakash et al., 2014 [20]
k_rec_ (h^−1^)	Recycling rate constant of ASGPR	13.8	Schwartz et al., 1982 [19]
k_deg.C_ (h^−1^)	siRNA degradation rate constant in cytoplasm	0.10 ^a^	Fixed
RISC_tot_ (μmol/L)	Total RISC concentration	0.0003	Wang et al., 2012 [21]
K_off.RISC_ (h^−1^)	Dissociation rate constant of siRNA antisense strand and RISC	1 × 10^−7^	Barlett and Davis 2006 [22]
k_DR_ (h^−1^)	Degradation rate constant of RISC complex	0.0033	Optimized
**Global PBPK Parameters**			
**Parameter (Unit)**	**Description**	**Value**	**Reference**
ka (h^−1^)	Absorption rate constant	0.84	Optimized
fu	Fraction of free GalNAc-siRNA in plasma	1.00	Optimized
P_liver_ (cm/min)	Endothelial permeability	0.02	Optimized
Kp	Partition coefficient between plasma and interstitial space	0.94	Niederalt et al., 2018 [13]
k_uptake_ (min^−1^)	Global endosomal uptake rate in remaining tissue	20.87	Optimized
k_recycling_ (min^−1^)	Global endosomal recycling rate constant in remaining tissue	0.000077	Optimized
k_kid.uptake_ (min^−1^)	Kidney endosomal uptake rate constant in remaining tissue	68.2	Optimized
k_kid.recycling_ (min^−1^)	Kidney endosomal recycling rate constant in remaining tissue	0.00039	Optimized
k_RNase_ (h^−1^)	Ribonuclease degradation rate constant	0.00012	Optimized
RNase_kidney_ (μmol/L)	Ribonuclease concentration in kidney tissue	1.17	Optimized
RNase_remaining_ (μmol/L)	Ribonuclease concentration in remaining tissue	0.17	Optimized

^a^ Fixed to the literature value to avoid overparameterization, Ayyar et al., 2021 [8].

**Table 3:** Summary of compound-specific WB-PBPK-PD parameters.

Parameter	Description	ALN-AT3/SIAT-2 ^a^ (1–25 mg/kg) ESC ^e^	SITTR-1/2 ^b^ (0.5–10 mg/kg) ESC ^e^	siF7-1 ^c^ (2.5 mg/kg) ESC ^e^	siF7-2/3 ^c^ (0.75–1 mg/kg) ADV ESC ^f^	siF9-1 ^d^ (2.5 mg/kg) ESC ^e^	siF9-2 ^d^ (0.75 mg/kg) ADV ESC ^f^
**Compound-Specific PBPK Parameters**							
F (%)	Bioavailability	40.1/73.0	63.2	10.2	19.5	5.0	10.3
k_endosome_ (1/h)	Endosomal degradation rate for siRNA	0.012	0.042	0.010	0.0066	0.0066	0.0042
k_on.RISC_ (L/nmol/h)	Association rate constant of siRNA antisense strand and RIS	0.00027				0.0014	
**Pharmacodynamic Parameters**							
**S_max_**	Maximum stimulation of mRNA degradation	13.1	140.2	85.5		40.5	
SC_50_ (nmol/L)	RISC-loaded siRNA at half-maximal stimulation	3.52	4.07	1.71			
Gamma	Gamma coefficient for target protein knockdown	1.5 ^g^	0.42	1.5 ^g^			
k_deg.mRNA_ (1/h)	Degradation rate constant for mRNA	0.06 ^g^					
k_deg.Protein_ (1/h)	Degradation rate target for protein	0.05 ^g^					

^a^ GalNAc-siRNA targeting antithrombin; ^b^ GalNAc-siRNA targeting transthyretin protein; ^c^ GalNAc-siRNA targeting Coagulation Factor VII; ^d^ GalNAc-siRNA targeting Coagulation Factor IX; ^e^: enhanced stabilization chemistry; ^f^ advanced enhanced stabilization chemistry; and ^g^ Ayyar et al., 2021 [8].

## Data Availability

The data presented in this study are available in this article and Supplementary Materials.

## Supplementary Materials

The following supporting information can be downloaded at https://www.mdpi.com/article/10.3390/pharmaceutics17010069/s1, Figure S1: Overview of the calculated average fold error (AFE) and absolute average fold error (AAFE) of the geometric mean of the simulated AUC_sim_ and observed AUC_obs_ for each measurement; Figure S2: Model simulations of N-acetyl galactosamine conjugated small interfering RNA (GalNAc-siRNA) in plasma, liver interstitial space and liver tissue vs. observed data with generic implementations of the two-pore formalism, no additional extravasation mechanism in the liver tissue together with previously reported mPBPK model parameters; Figure S3: Observed data for liver tissue concentrations dose normalized from 0–1000 h to show the difference in the terminal liver elimination phase (250–1000 h); Table S1: Summary of the observed area under the curve (AUC_obs_) and simulated area under the curve (AUC_sim_) for each compound and the AUC_sim_/AUC_obs_ ratios given as the fold change for each compound of their available measurements; Supplementary model file: WB-PBPK-PD_ALN-AT3_1 mg_kg_Case_Example.
