# Supplementary material for: Whole-Body Physiologically Based Pharmacokinetic Modeling of GalNAc-Conjugated siRNAs
Source: Pharmaceutics. 2025 Jan 6;17(1):69. doi: 10.3390/pharmaceutics17010069 (PMC11769416; doi:10.3390/pharmaceutics17010069)
Supplement: Supplementary file 1 [file pharmaceutics-17-00069-s001.zip › pharmaceutics-3382948-supplementary.pdf]

Whole-Body Physiologically Based Pharmacokinetic Modeling of GalNAc-Conjugated siRNAs

Emilie Langeskov Salim [1,2], Kim Kristensen [2] and Erik Sjögren[1,*]

[1] Department of Pharmaceutical Bioscience, Translational Drug Discovery and Development, Uppsala University, SE-75124 Uppsala, Sweden; emlv@novonordisk.com
[2] Department of Discovery PKPD & QSP Modelling, Novo Nordisk A/S, DK-2760 Måløv, Denmark; kkri@novonordisk.com

* Correspondence: erik.sjogren@uu.se; Tel.: +46-18-471-41-54

Supplementary S1

TMDD model differential equations.

$$\frac{dDA_{endosome}}{dt} = k_{int} \cdot DA - k_{cle} \cdot DA_{endosome} \quad \text{Eq. S1}$$

$$\frac{dA_{endosome}}{dt} = k_{cle} \cdot DA_{endosome} - k_{degA} \cdot A_{endosome} - k_{rec} \cdot A_{endosome} \quad \text{Eq. S2}$$

$$\frac{dD_{endosome}}{dt} = k_{cle} \cdot DA_{endosome} - k_{degC} \cdot D_{endosome} - k_{endosome} \cdot D_{endosome} \quad \text{Eq. S3}$$

$$\frac{dD_{cytoplasma}}{dt} = f_{escape} \cdot k_{endosome} \cdot D_{endosome} - k_{degC} \cdot D_{cytoplasm} - k_{onRISC} \cdot D_{cytoplasm} \cdot (RISC_{tot} - DRISC) + k_{off_{RISC}} \cdot DRISC \quad \text{Eq. S4}$$

$$\frac{dDRISC}{dt} = k_{onRISC} \cdot D_{cytoplasm} \cdot (RISC_{tot} - DRISC) - k_{off} \cdot DRISC - k_{DR} \cdot D_{cytoplasm} \quad \text{Eq. S5}$$

Where $DA_{endosome}$ is bound ASGPR-GalNAc-siRNA complex in in the endosomal compartment; DA is bound ASGPR-GalNAc-siRNA complex in the interstitial compartment; k_{int} is internalization rate constant of GalNAc-siRNA-ASGPR complex; k_{cle} is cleavage rate constant of GalNAc-siRNA in the endosomal compartment; k_{degA} degradation rate constant of ASGPR in cytoplasm; $A_{cytoplasm}$ is the free fraction of ASGPR in the cytoplasm; k_{degC} is the siRNA degradation rate constant in cytoplasm; $D_{endosome}$ is the free fraction of siRNA in the endosomal compartment, $k_{endosome}$ is the endosomal degradation and escape rate; f_{escape} is the fraction of siRNA escaping from the endosomal compartment to induce RISC formation; $D_{cytoplasm}$ is the fraction of free siRNA in the cytoplasm; k_{onRISC} is the association rate constant of siRNA antisense strand and RISC; $RISC_{tot}$ is the total RISC concentration; $DRISC$ is the bound RISC and siRNA complex; k_{DR} is degradation rate constant of RISC complex.

Supplementary S2

Area Under the Curve Calculation

The AUC_{obs} was compared to the AUC_{sim} by calculating the fold change given as the ratio between AUC_{obs} and AUC_{sim} as described in equation S7:

$$Fold\ Change = \frac{AUC_{sim}}{AUC_{obs}} \qquad \text{Eq. S6}$$

Table S1. Summary of the observed area under the curve (AUC_{obs}) and simulated area under the curve (AUC_{sim}) for each compound and the AUC_{sim}/AUC_{obs} ratios given as the fold change for each compound of their available measurements.

Compound	Dose/Administration/Design	Measurement	AUC_{sim} (ug·h/mL)	AUC_{obs} (ug·h/mL)	Fold Change (AUC_{sim}/AUC_{obs})
ALN-AT3	1 mg/kg SC, ESC	Plasma	0.09	0.17	0.52
ALN-AT3	2.5 mg/kg SC, ESC	Plasma	0.22	0.37	0.60
ALN-AT3	5 mg/kg SC, ESC	Plasma	0.45	0.67	0.67
SIAT-2	25 mg/kg SC, ESC	Plasma	11.58	9.45	1.22
SITTR-2	10 mg/kg SC, ESC	Plasma	2.02	2.61	0.78
SITTR-2	10 mg/kg IV, ESC	Plasma	2.17	2.89	0.75
ALN-AT3	1 mg/kg SC, ESC	Liver	411.77	292.61	1.41
ALN-AT3	2.5 mg/kg SC, ESC	Liver	1028.06	796.61	1.29
ALN-AT3	5 mg/kg SC, ESC	Liver	2049.96	2088.87	0.98
SIAT-2	25 mg/kg SC, ESC	Liver	11813.51	20041.15	0.59
SITTR-2	10 mg/kg SC, ESC	Liver	1888.44	2106.98	0.90
SITTR-2	10 mg/kg IV, ESC	Liver	897.70	1251.78	0.72

SIF7-1	2.5 mg/kg SC, ESC	Liver	293.46	263.09	1.12
SIF7-2	0.75 mg/kg SC, ADV ESC	Liver	331.67	161.91	2.05
SIF7-3	1 mg/kg SC, ADV ESC	Liver	338.90	333.11	1.01
SIF9-1	2.5 mg/kg SC, ESC	Liver	250.47	310.78	0.81
SIF9-2	0.75mg/kg SC, ADV ESC	Liver	355.09	200.58	1.77
SITTR-2	10 mg/kg SC, ESC	Kidney	148.63	230.53	1.42
SITTR-2	10 mg/kg IV, ESC	Kidney	592.99	416.18	0.64
SIAT-2	2.5 mg/kg SC, ESC	RISC	1.57	1.73	0.90
SIF7-1	2.5 mg/kg SC, ESC	RISC	0.42	0.17	2.44
SIF7-2	0.75mg/kg SC, ADV ESC	RISC	0.23	0.17	1.29
SIF7-3	1 mg/kg SC, ADV ESC	RISC	0.17	0.11	1.59
SIF9-1	2.5 mg/kg SC, ESC	RISC	0.81	1.00	0.80
SIF9-2	0.75mg/kg SC, ADV ESC	RISC	0.62	0.42	1.29

The model was further quantitatively evaluated by calculation of the average fold error (AFE) and absolute average fold error (AAFE) for each measurement as described in equation S7 and S8:

$$average\ fold\ error = 10^{\sum log\left(\frac{AUC_{sim}}{AUC_{obs}}\right)/n} \quad \text{Eq. S7}$$

$$absolute\ average\ fold\ error = 10^{\sum \left|log\left(\frac{AUC_{sim}}{AUC_{obs}}\right)\right|/n} \quad \text{Eq. S8}$$

Where AUC_{sim} is the simulated AUC; AUC_{obs} is the observed AUC; n is the number of observations of the respective measurement.

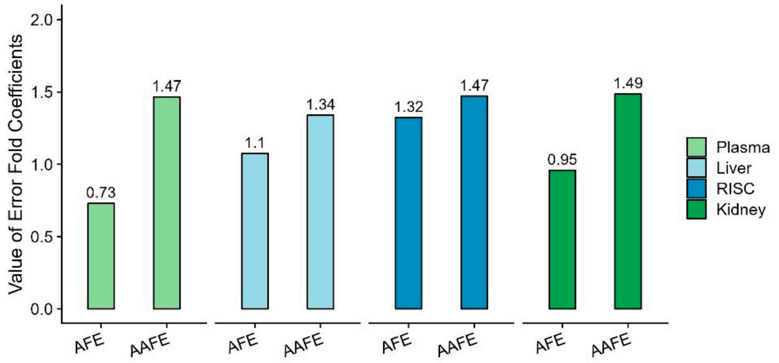

Figure S1. Overview of the calculated average fold error (AFE) and absolute average fold error (AAFE) of the geometric mean of the simulated AUC_{sim} and observed AUC_{obs} for each measurement.

Supplementary S3

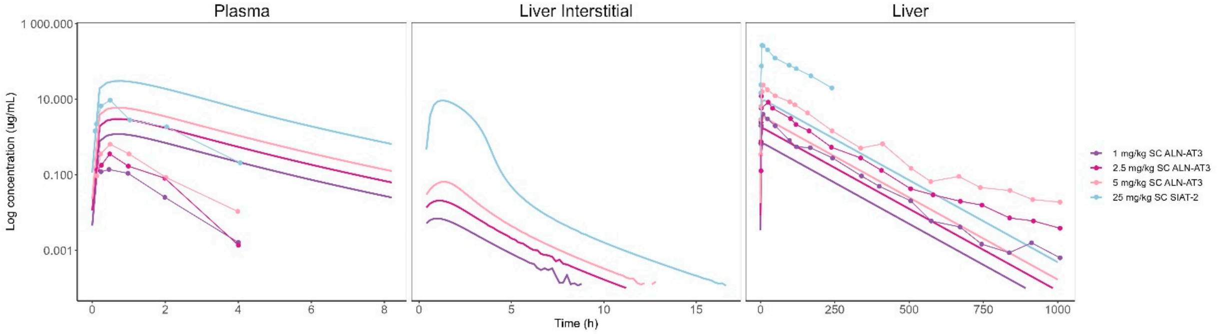

Figure S2. Model simulations of N-acetyl galactosamine conjugated small interfering RNA (GalNAc-siRNA) in plasma, liver interstitial space and liver tissue vs. observed data with generic implementations of the two-pore formalism, no additional extravasation mechanism in the liver tissue together with previously reported mPBPK model parameters. Subsequently prolonging the circulation in the plasma, restricting the amount of GalNAc-siRNA in the liver interstitial space and consequently underpredicting ASGPR mediated uptake of GalNAc-siRNA the liver tissue. Solid lines represent model simulations of GalNAc-siRNAs, ALN-AT3/SIAT-2, targeting antithrombin and dots represent observations of ALN-AT3/SIAT-2. Dark purple line represents 1 mg/kg subcutaneous administered ALN-AT3; Dark pink represents 2.5 mg/kg administered subcutaneous ALN-AT3; Light pink line represents 5 mg/kg administered subcutaneous ALN-AT3; Light blue line represents 25 mg/kg subcutaneous administered SIAT-2.

Supplementary S4

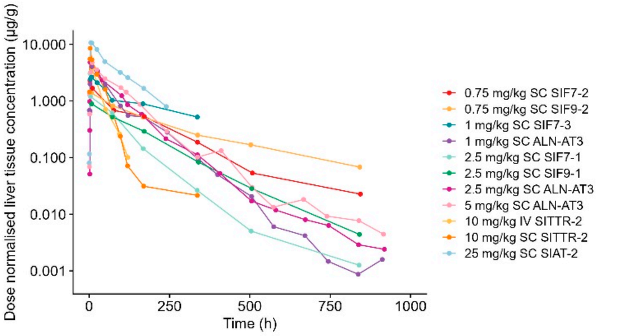

Figure S3. Observed data for liver tissue concentrations dose normalized from 0–1000 h to show the difference in the terminal liver elimination phase (250–1000 h). Dots represents observed datapoints. Dark purple line represents 1 mg/kg subcutaneous ALN-AT3; Dark pink represents 2.5 mg/kg administered subcutaneous ALN-AT3; Light pink line represents 5 mg/kg administered subcutaneous ALN-AT3; Light blue line represents 25 mg/kg subcutaneous administered SIAT-2. B) Orange line represents 10 mg/kg subcutaneous administered SITTR2; Yellow line represents 10 mg/kg intravenously administered SITTR2. C) Red line represents 0.75 mg/kg subcutaneous administered SIF72; Blue line represents 1 mg/kg subcutaneous administered SIF73; Light green line represents 2.5 mg/kg subcutaneous administered SIF71. D) Light orange line represents 0.75 mg/kg subcutaneous administered SIF92; Green line represents 2.5 mg/kg subcutaneous administered SIF91.
